# Caspofungin for post solid organ transplant invasive fungal disease: results of a retrospective observational study

**DOI:** 10.1111/j.1399-3062.2009.00490.x

**Published:** 2010-06

**Authors:** M Winkler, J Pratschke, U Schulz, S Zheng, M Zhang, W Li, M Lu, D Sgarabotto, G Sganga, P Kaskel, S Chandwani, L Ma, J Petrovic, M Shivaprakash

**Affiliations:** 1 Medizinische Hochschule Hannover, Klinik für Allgemein-, Viszeral- und TransplanationschirurgieHannover, Germany; 2 Charité– Universitätsmedizin Berlin, Klinik für Viszeral- und TransplantationsmedizinBerlin, Germany; 3 Herz- und Diabeteszentrum Nordrhein-Westfalen, Ruhr-Universität Bochum, Klinik für Thorax- und KardiovaskularchirurgieBad Oeynhausen, Germany; 4 Zhejiang UniversityZhejiang, China; 5 Beijing Chao-Yang HospitalBeijing, China; 6 Hospital of Sun-Yat Sen UniversityGuangzhou, China; 7 Ospedale Civile di PadovaPadova, Italy; 8 Department of Surgery, Division of General Surgery and Organ Transplantation, Catholic UniversityRome, Italy; 9 Department of Outcomes Research, MSD Sharp & Dohme GmbHHaar, Germany; 10 University of Medicine and Dentistry of New Jersey, SomersetNew Jersey, USA; 11 Outcomes Research, Merck & Co. Inc., Whitehouse StationNew Jersey, USA; 12 Merck Sharp & Dohme Italia S.p.A.Rome, Italy

**Keywords:** aspergillosis, candidiasis, caspofungin, solid organ transplant, invasive fungal disease

## Abstract

M. Winkler, J. Pratschke, U. Schulz, S. Zheng, M. Zhang, W. Li, M. Lu, D. Sgarabotto, G. Sganga, P. Kaskel, S. Chandwani, L. Ma, J. Petrovic, M. Shivaprakash. Caspofungin for post solid organ transplant invasive fungal disease: results of a retrospective observational study. Transpl Infect Dis 2010: **12:** 230–237. All rights reserved

**Objective:**

This study was designed to determine clinical outcomes with caspofungin in patients with proven or probable invasive fungal infection (IFI) after a solid organ transplant (SOT) procedure.

**Methods:**

In this retrospective observational study, data were collected for a single episode of IFI in patients with an SOT between January 2004 and June 2007. Response was determined by the investigator as favorable (complete or partial) or unfavorable (stable disease or failure) at the end of caspofungin therapy (EOCT). The primary effectiveness population was the proportion of patients who received ≥5 doses of caspofungin (modified all-patients-treated population). Safety was assessed for patients who received ≥1 dose of caspofungin.

**Results:**

A total 81 of patients from 13 sites in China, Germany, Italy, and the United Kingdom were enrolled, including 49 (60%) liver, 22 (27%) heart, 5 (6%) lung, 2 (2%) kidney, 2 (2%) liver and kidney, and 1 (1%) pancreas and kidney recipients. Candidiasis was diagnosed in 64/81 patients (79%) and aspergillosis in 22/81 patients (27%). Most patients received caspofungin monotherapy (75%). Caspofungin was given as first-line therapy to 59 (73%) patients. The overall favorable response at EOCT was 87% (58/67; 95% confidence interval [CI]: 76%, 94%), with favorable responses in 88% (43/49; 95% CI: 75%, 95%) of patients receiving caspofungin monotherapy and 83% (15/18; 95% CI: 59%, 96%) of patients receiving combination therapy with caspofungin (modified all-patients-treated population). Response by type of SOT was as follows: liver 87% (39/45), heart 93% (14/15), kidney 100% (5/5), and lung 50% (2/4). An overall survival rate (all-patients-treated) of 69% (56/81; 95% CI: 59%, 79%) was observed at 7 days post EOCT. No serious drug-related adverse events were reported.

**Conclusion:**

In this study, caspofungin was effective and well tolerated in the treatment of IFIs involving SOT recipients.

Fungal infections are a common cause of morbidity and mortality in solid organ transplant (SOT) recipients. The reported incidence of fungal infections ranges from 4–42% in liver transplant recipients (1) and from 15–35% in lung transplant recipients (2). *Candida* species are the most common pathogens, accounting for approximately 43–80% of fungal infections following liver (1, 3), heart (4), and lung transplantation (5). *Aspergillus* species have been isolated from approximately 9–34% of patients with invasive fungal infection (IFI) after liver transplantation (6) and 20–50% after lung transplantation (5–7). Increased mortality rates (20–90%) have been reported among SOT recipients suffering from IFIs compared with SOT patients with no IFI (6, 8, 9).

Risk factors for *Candida* infection include a high intraoperative transfusion requirement as well as post-transplant bacterial infection (1). Renal failure and the need for dialysis have been described as risk factors for early-onset *Aspergillus* infection; risk factors in patients with late-onset infections (>3 months after transplantation) included age, intensified immunosuppression due to factors such as chronic transplant rejection, and post-transplant renal failure (10). While optimizing treatment of IFI in SOT patients may decrease attributable mortality (6, 11), limited data are available from randomized clinical trials regarding which treatments are most effective for SOT patients with IFI (12). Current therapeutic options for invasive fungal disease include echinocandins, polyenes, and azoles (13–16).

Caspofungin is an echinocandin approved for treating adult and pediatric patients with invasive candidiasis, as empirical therapy in presumed fungal infections including *Candida* or *Aspergillus* in febrile, neutropenic adult patients, and for treatment of invasive aspergillosis in adults who are refractory or intolerant to other antifungal agents. It is metabolized independently of cytochrome 450 and may therefore have fewer interactions with calcineurin inhibitors (CNIs) such as cyclosporine or tacrolimus (17–19). This retrospective observational study evaluated the effectiveness and safety of caspofungin as treatment for invasive fungal disease in patients who had received an SOT.

## Patients and methods

This retrospective, multicenter observational study was designed to evaluate clinical and safety outcomes in patients with proven or probable invasive fungal disease treated with caspofungin following a SOT. The study was performed at 13 transplant centers in China (*n*=3), Germany (*n*=5), Italy (*n*=4), and the United Kingdom (*n*=1). Central and local regulatory and Independent Ethics Committee approvals were obtained as required by each site or country.

Patients who received an SOT between January 2004 and January 2007 and had received caspofungin following SOT for treatment of a proven or probable IFI were eligible for inclusion. EORTC-MSG criteria were used as a guide to assess whether IFI cases were proven or probable (20). Charts were reviewed between July 2007 and December 2007. If multiple transplant procedures took place, the procedure closest in time to the onset of post-transplant IFI was considered. Patients who participated in Merck-sponsored clinical studies for IFI during the hospitalization were excluded. As this was a retrospective observational study, no medication was provided to sites. Caspofungin was administered according to clinician's judgment and local diagnostic and treatment pathways.

### Effectiveness and safety assessments

Data for consecutive patient cases meeting the eligibility criteria were entered at each site into this observational study. Internet-based electronic case report forms were used to collect patient data. Demographic characteristics, medical history, caspofungin dosing regimen, other antifungal drugs received, co-administered immunosuppressive agents, and dose and duration of therapy were recorded. Information was collected on co-morbidities and predisposing risk factors, i.e., active malignancy, renal failure requiring dialysis, primary graft non-function, re-transplantation, exposure to >3 antibiotics, recent use of central venous catheter, current steroid dose (i.e., during this hospitalization), current monoclonal antibody use for immunosuppression (i.e., during this hospitalization), hyperglycemia, neutropenia (absolute neutrophil count <500 cells/μL), recent parenteral nutrition (hyperalimentation), United Network for Organ Sharing (UNOS) Class 1 (i.e., patients with a life expectancy of <7 days without a liver transplant and with fulminant [sudden] liver failure, or with newly transplanted liver not functioning, respectively [21]) clinical urgency of transplantation, pretransplantation fungal colonization, cytomegalovirus (CMV) infection, long duration of transplant procedure (>5 h), biliary construction using Roux loop, re-operation (laparatomy) within 5 days after transplantation, need for substantial (≥40 U) intraoperative transfusions, hepatic iron overload, thrombocytopenia, fulminant hepatic failure, recent intensive care unit (ICU) stay (i.e., during this hospitalization), and ambient/community-acquired exposure (i.e., before hospitalization). Data on type of SOT, site of IFI following transplantation, mycology, and severity of illness measures were also collected.

According to the pre-specified analysis plan, the primary effectiveness population was based on a modified all-patients-treated population, which included all patients who received ≥5 doses of caspofungin for treatment of IFI. Mycological information, if available, was collected via chart review and included site of fungal infection, type of fungal infection, diagnostic tests used to identify fungal species, and diagnosis of the infection. As part of the chart review, no *post hoc* verification of mycological tests and no resistance tests were performed. The chart review involved taking data as available in the charts.

Safety was assessed for the all-patients-treated population, which was defined as any patient who received at least 1 dose of caspofungin. A summary of the number and percentage of patients who had at least 1 drug-related adverse event leading to early discontinuation of caspofungin or death was produced by collecting drug-related clinical and laboratory adverse events, drug–drug interactions, and discontinuations or deaths associated with drug-related adverse events. Drug-related events referred to those events considered by the investigator as possibly, probably, or definitely related to caspofungin therapy. All outcomes were determined based on the judgment of the investigator.

### Definitions of response

The primary endpoint was the proportion of patients who received ≥5 doses of caspofungin and had a favorable response to treatment. Treatment response was determined by the investigator as favorable (complete or partial) at the end of caspofungin therapy (EOCT), or unfavorable (stable disease or disease progression) at EOCT. In addition, survival was assessed at hospital discharge.

Complete response was defined as resolution of all attributable clinical and radiological signs and symptoms of invasive mycosis at EOCT; partial response was defined as a substantial reduction of attributable clinical and radiological pre-treatment signs and symptoms of invasive mycosis at EOCT; stable disease was defined as minimal or no reduction of attributable clinical and radiological signs and symptoms of invasive mycosis at EOCT; and failure was defined as worsening of signs and symptoms of invasive mycosis at EOCT. Response was determined based on the treating physician's clinical judgment. Minimal observation period was 7 days after EOCT.

### Statistical analysis

Patient characteristics, patient risk factors for invasive fungal disease, indication for caspofungin therapy, immunosuppressive therapy at onset of caspofungin therapy, and proportion of favorable response by pathogen were assessed overall, and by type of therapy (caspofungin monotherapy and caspofungin combination therapy, respectively) using descriptive statistics.

## Results

### Patient baseline characteristics

A total of 81 patients were included in this observational study. Sixty-one patients (75%) were male. Patients had a median of 2 medical comorbidities at study entry (mean 2.8; range 1–8); liver disease was the most common condition (52/81 patients, 64). SOTs included liver in 49 patients (60%), heart in 22 patients (27%), lung in 5 patients (6%), kidney in 2 patients (2%), liver and kidney in 2 patients (2%), and pancreas and kidney in 1 patient (1%). At initiation of caspofungin therapy, the median age was 54 years (range 24–70; standard deviation [SD], 10.38), and the median weight was 68 kg (range 44–103 kg; SD, 12.95). The median duration of transplant operation was 6.5 h (SD, 3.551).

### Risk factors and immunosuppression

Patients had a median of 8 risk factors for IFI at initiation of caspofungin therapy (Table 1). Patient risk factors included recent use of central venous catheter (90%), recent ICU stay (85%), current steroid dose (83%), duration of transplant procedure >5 h (70%), exposure to >3 antibiotics (53%), and recent parenteral nutrition (53%). At the start of caspofungin therapy, 76/81 patients (94%) were non-neutropenic, 49/81 patients (61%) had mechanical ventilation, 29/81 patients (36%) had renal replacement therapy, and 16/81 (20%) had evidence of acute 14/16 (88%) or chronic 2/16 (13%) organ rejection.

**Table 1:** **Patient risk factors for invasive fungal disease with at least 1 patient affected**

	*N* (%)		
	Caspofungin monotherapy^1^*N*=61	Combination therapy *N*=20	Overall *N*=81
Number of risk factors per patient^2^ (median [mean; range; SD])	8 [8.2; 4–18; 2.6]	8 [7.3; 2–15; 3.4]	8 [8.0; 2–18; 2.9]
Active malignancy	5 (8)	4 (20)	9 (11)
Renal failure requiring dialysis	25 (41)	5 (25)	30 (37)
Primary graft non-function	2 (3)	3 (15)	5 (6)
Re-transplantation	5 (8)	4 (20)	9 (11)
Exposure to >3 antibiotics	29 (48)	14 (70)	43 (53)
Recent use of central venous catheter	55 (90)	18 (90)	73 (90)^2^
Current steroid dose^3^	50 (82)	17 (85)	67 (83)
Current monoclonal antibody use for immunosuppression^3^	21 (34)	4 (20)	25 (31)
Hyperglycemia	23 (28)^2^	11 (55)	34 (42)
Neutropenia^4^	4 (7)	0 (0)	4 (5)
Recent parenteral nutrition (hyperalimentation)	36 (59)	7 (35)	43 (53)
UNOS Class 1	14 (23)	2 (10)	16 (20)
Clinical urgency of transplantation	19 (31)	3 (15)	22 (27)
Pretransplantation fungal colonization	8 (13)	2 (10)	10 (12)
CMV infection	15 (25)	5 (25)	20 (25)
Long duration of transplant procedure (>5 h)	47 (77)	10 (50)	57 (70)
Biliary construction using Roux loop	7 (12)	1 (5)	8 (10)
Re-operation (laparatomy) within 5 days after transplantation	11 (18)	5 (25)	16 (20)
Need for substantial (≥40 U) intraoperative transfusions	28 (46)	3 (15)	31 (38)
Thrombocytopenia	15 (25)	7 (35)	22 (27)
Fulminant hepatic failure	5 (8)	1 (5)	6 (7)
Recent ICU stay^3^	54 (89)	15 (75)	69 (85)
Ambiental/community acquired exposure (i.e., before hospitalization)	2 (3)	2 (10)	4 (5)
Other	1 (1.6)	0 (0)	1 (1.2)

1 Data for 1 patient in the monotherapy group were missing except for the categories designated ‘Other,’‘Biliary construction using Roux loop,’ and ‘CMV infection.’

2 At initiation of caspofungin therapy.

3 During this hospitalization.

4 ANC<500 cells/μL.

SD, standard deviation; UNOS, United Network for Organ Sharing; CMV, cytomegalovirus; ANC, absolute neutrophil count; ICU, intensive care unit.

Overall, 71/81 patients (88%) received immunosuppressive therapy (Table 2). Corticosteroids were used by 65/81 patients (80%; mainly prednisolone [median dose 25 mg/day; mean 62.8; range 5–750; *n*=28] and prednisone [median dose 10 mg/day; mean 10; range 5–750; *n*=13]). Tacrolimus, administered to 54% of patients; mycophenolate mofetil, administered to 27%; and cyclosporin A, administered to 25% of patients, were among the most frequently prescribed immunosuppressants. Compared with caspofungin combination therapy, patients on caspofungin monotherapy were numerically more likely to have a history of substantial intraoperative transfusions (prevalence ratio [PR] 3.1, *P=*0.02 [Fisher's exact]), UNOS Class 1 (PR 2.3, *P=*0.2), biliary Roux loop construction (PR 2.3, *P=*0.7), or clinical urgency of transplantation (PR 2.1, *P*=0.2), and were numerically less likely to have primary graft non-function (PR 0.2, *P*=0.1), ambient/community acquired fungal infection (PR 0.3, *P*=0.3), re-transplantation (PR 0.4, *P*=0.2), or active malignancy (PR 0.4, *P*=0.2).

**Table 2:** **Immunosuppressive therapy at onset of caspofungin therapy**

	Caspofungin monotherapy		Combination therapy		
Immunosuppressive therapy^1^	With steroid (*N*=47)	Without steroid (*N*=14)	With steroid (*N*=18)	Without steroid (*N*=2)	Overall (*N*=81)
Any therapy	43 (91.5)	10 (71.4)	18 (100)	0 (0.0)	71 (87.7)
Tacrolimus	25 (53.2)	10 (71.4)	9 (50.0)	0 (0.0)	44 (54.3)
Cyclosporin A	12 (25.5)	0 (0.0)	8 (44.4)	0 (0.0)	20 (24.7)
Sirolimus	0 (0.0)	0 (0.0)	1 (5.6)	0 (0.0)	1 (1.2)
Mycophenolate mofetil	21 (44.7)	2 (14.3)	3 (16.7)	0 (0.0)	26 (32.1)
Azathioprine	1 (2.1)	0 (0.0)	1 (5.6)	0 (0.0)	2 (2.5)
Other	6 (12.7)	3 (21.4)	2 (11.1)	0 (0.0)	11 (13.6)
Basiliximab	1 (2.1)	2 (14.3)	1 (5.5)	0 (0.0)	4 (4.9)
Muromonab-CD 3	0 (0.0)	0 (0.0)	1 (5.5)	0 (0.0)	1 (1.2)
Daclizumab	3 (6.4)	1 (7.1)	0 (0.0)	0 (0.0)	4 (4.9)

1 Patients may have received >1 immunosuppressive therapy.

### Description of infection

A total of 66/81 patients were treated for IFI during the initial phase following SOT; 15/81 patients (19%) had been discharged from the hospital following transplant but before IFI. At least 1 culture was taken from sterile sites for 47/81 patients (58.0%), with 21 from the lung. Cultures from non-sterile sites were taken from 43/81 patients (53.1%), in particular from respiratory secretions in 34/43 cases (79%). Proven fungal infections were diagnosed in 38/81 patients (46.9%), with 9/38 due to proven *Aspergillus* infection (23.7%), 23/38 to proven *Candida* infection (60.5%), 5/38 (13.2%) mixed infections, and 1/38 (2.6%) due to a mold infection in a surgical wound in an Asian patient that could not be specified further (Table 3). The site of infection was the lung in 50/81 patients (62%), multiple sites in 13/81 patients (16%), blood in 6/81 (7%), sinus in 2/81 (3%), and intra-abdominal infection in 6/81 (7%; organs afflicted: abdomen not further specified 3, kidney 1, liver/spleen 1, and abscess not further specified 1). Four patients had other sites of infection (5%).

**Table 3:** **Indication for caspofungin therapy fungal infection**

	Caspofungin monotherapy *N*=61	Combination therapy *N*=20	Overall *N*=81
*Proven fungal infection (n=38/81)*			
Aspergillosis	4 (14)	5 (56)	9 (24)
Candidiasis	22 (76)	1 (11)	23 (61)
Mixed^1^	2 (7)	3 (33)	5 (13)
Other^2^	1 (3)	0 (0)	1 (3)
*Probable fungal infection (n=43/81)*			
Aspergillosis	5 (16)	2 (18)	7 (16)
Candidiasis	26 (81)	9 (82)	35 (81)
Mixed^1^	1 (3)	0 (0)	1 (2)

1 Mixed category indicates *Aspergillus* and *Candida* infection.

2 Other category includes other mold infection.

### Prior or adjuvant antifungal therapy

All patients were treated with caspofungin either as first-line (*n*=59) or second-line (*n*=22) therapy. At the discretion of the treating physician, and based on local diagnostic and treatment pathways only, caspofungin was given as monotherapy to 61 patients; the remaining 20 patients received concomitant therapy with other antifungal agents.

Before initiation of caspofungin, 30 patients had been treated with other antifungals. Of these, 10 had received prophylactic treatment (1 amphotericin B colloid dispersion, 6 fluconazole, 1 voriconazole, 2 itraconazole); 15 empiric therapy (2 liposomal amphotericin B, 3 voriconazole, 6 fluconazole, 1 amphotericin B colloid dispersion/voriconazole, 1 itraconazole, 2 voriconazole/fluconazole combination); and 5 definite therapy (1 fluconazole, 1 amphotericin lipid complex, 1 fluconazole/voriconazole, 1 fluconazole/voriconazole/liposomal amphotericin B, 1 itraconazole/fluconazole). Reason for switching to or adding caspofungin was immunosuppressive therapy in 10 patients; clinically refractory to first-line antifungal in 8; microbiologically refractory to first-line antifungal in 3; nephrotoxicity in 2; sensitivity to caspofungin in 2; and unknown in 5. Of the 20/67 patients who received combination therapy, in addition to caspofungin, 10% received voriconazole; 9% received amphotericin B or its lipid formulations; and 1% received voriconazole and fluconazole. Treatment was initiated for proven fungal infection in 38/81 patients (47%) and probable fungal infection in 43/81 patients (53%), respectively (Table 3). Following caspofungin therapy, 15 (19%) patients received fluconazole, 10 (12%) received voriconazole, 4 (5%) received amphotericin B or its lipid formulations, and 10 (12%) received other antifungals.

### Clinical response

A favorable (complete or partial) response at EOCT was observed in 58/67 patients (87%; 95% confidence interval [CI] 76%; 94%) in the modified all-patients-treated population. In the modified all-patients-treated population, 35/67 patients (52%) had a complete response, and 23/67 (34%) had a partial response. A favorable response was seen in 67/81 patients (83%; 95% CI 73%; 90%) in the all-patients-treated population. Favorable responses were also noted in 14/19 patients with probable or proven *Aspergillus* infection (74%), and in 59/66 patients with *Candida* infection (89%; Table 4).

**Table 4:** **Proportion of patients with favorable response by pathogen**

Pathogen	Caspofungin monotherapy *n*/*N* (% [95% CI])	Combination therapy *n*/*N* (% [95% CI])	Total *n*/*N* (% [95% CI])
*Aspergillus*	7/9 (78 [40; 97])	7/10 (70 [35; 93])	14/19 (74[49; 91])
*Candida albicans*	24/26 (92 [75; 99])	8/10 (80 [44; 98])	32/36 (89 [74;97])
Non-*albicans Candida*	19/20 (95 [75; 100])	4/5 (80 [28; 100])	23/25 (92[74; 99])
Mixed *Candida* species	2/3 (67 [9; 99])	2/2 (100 [16; 100])	4/5 (80 [28; 100])

CI, confidence interval.

### Safety

In the all-patients-treated population, 25/81 (30.9%) patients had died at the end of observation, i.e., hospital discharge. According to the individual physician's judgment, fungal infection was the primary reason for death in 3/81 patients (3.7%). Other reasons for death were multi-organ failure (*n*=12, 14.8%); single organ failure (*n*=4, 4.9%); bacterial infection (*n*=3, 3.7%); graft-versus-host disease in a patient with photo skin type VI who had received a liver transplant for cryptogenic cirrhosis, suffered from neutropenia, and had received treatment with monoclonal antibodies (*n*=1, 1.2%); bronchopneumonia and acute respiratory distress syndrome (*n*=1, 1.2%); and diffuse hemorrhage (*n*=1, 1.2%). One patient in the caspofungin monotherapy group experienced drug-related adverse events (pyrexia and hyperbilirubinemia) as did 1 in the combination therapy group (nausea). No serious drug-related adverse events were reported. No patients required discontinuation of caspofungin due to a drug-related adverse event. In the modified all-patients-treated population, 18/67 patients (26.9%) died at the end of observation, among these 6 during the first 7 days after completion of caspofungin therapy.

## Discussion

Invasive fungal disease has become an important cause of death in SOT recipients, particularly following lung or liver transplantation. Amphotericin B was the only drug available for these patients for years, thus amphotericin B and its lipid formulations were often used to treat these patients despite the risk of hepatic failure (22) and renal impairment (23), which can be exacerbated when combined with CNIs (6). SOT recipients may in fact be excluded from large trials because of the potential confounding complications of mandatory immunosuppressive therapy. Drug interactions with CNIs are a major issue in transplant patients. The advent of newer agents such as the echinocandins may represent a therapeutic opportunity. Caspofungin is not associated with major drug-to-drug interactions involving the cytochrome 450 metabolism of CNIs (18). This factor was important in the present study, where 9 patients were switched to caspofungin from another antifungal agent owing to drug interactions with immunosuppressive therapy. Most patients who were effectively managed with caspofungin received concomitant immunosuppressive agents. These results are particularly relevant because current published data on caspofungin in SOT patients have been mostly in the form of anecdotal case reports (24–26), reports on small patient groups (27), and salvage studies (19).

In our study, *Candida* was the most common cause of fungal infection. Sites of infection were comparable to those reported in other studies (1, 9). We found a relatively higher number of *Candida* and *Aspergillus* mixed infections compared with the literature (6), although Fujishita et al. (28) reported 3 hemato-oncological cases of pulmonary mycosis due to mixed *Candida* and *Aspergillus* infection in 32 patients (9.4%). One possible explanation for the higher number of mixed infections is that many studies focus on either *Aspergillus* or *Candida* species while our study evaluated all eligible SOT recipients.

Caspofungin was effective as first- and second-line therapy in this group of patients with a favorable response rate of 87% in SOT recipients with proven or probable IFI. It was noteworthy that 89% of patients with proven or probable invasive *Candida* infection and 74% of patients with proven or probable *Aspergillus* infection responded to caspofungin treatment, similar to results reporting that caspofungin was an effective treatment in invasive aspergillosis after SOT (29). Prophylaxis for spontaneous bacterial peritonitis, fulminant hepatitis, retransplantation, dialysis, and CMV viremia have been identified as risk factors for invasive *Candida* infection in liver transplant recipients (30). Favorable response rates in our patients presenting these 5 risk factors were between 62.5% (retransplantation) and 85.7% (exposure to >3 antibiotics).

The overall mortality observed (31% at 7 days post EOCT) was relatively high. This is most likely because the patients included in this study were severely ill. The 24 patients with APACHE II score data had a median score of 23, where a score of >20 is often used to indicate high severity of illness. Also, a large majority of patients received immunosuppressive therapy. The mean duration of transplant operation of ∼7 h indicates more difficult operative procedures.

This study had several inherent limitations. As a retrospective observational study, there was no comparator arm. Also, because of potential confounding due to imbalances of prognostic factors, no conclusions should be drawn regarding the outcome of caspofungin monotherapy relative to the outcome of caspofungin combination therapy. Results may have been affected by the relatively low number of patients in some categories, and there was a relatively brief follow-up period after the EOCT.

Nevertheless, in this retrospective observational study, the clinical response rate of patients treated with caspofungin for IFI after SOT was comparable to rates shown in randomized trials in non-SOT patients.
